# IL-37 suppresses hepatocellular carcinoma growth by converting pSmad3 signaling from JNK/pSmad3L/c-Myc oncogenic signaling to pSmad3C/P21 tumor-suppressive signaling

**DOI:** 10.18632/oncotarget.13196

**Published:** 2016-11-08

**Authors:** Rui Liu, Chengyong Tang, Ai Shen, Huating Luo, Xufu Wei, Daofeng Zheng, Chao Sun, Zhongtang Li, Di Zhu, Tingting Li, Zhongjun Wu

**Affiliations:** ^1^ Department of Hepatobiliary Surgery, The First Affiliated Hospital of Chongqing Medical University, Chongqing 400016, China; ^2^ Department of Clinical Pharmacology, The First Affiliated Hospital of Chongqing Medical University, Chongqing 400016, China; ^3^ Department of Hepatobiliary Surgery, Chongqing Cancer Institute, Chongqing 400030, China; ^4^ Department of Oncology, The First Affiliated Hospital of Chongqing Medical University, Chongqing 400016, China

**Keywords:** hepatocellular carcinoma, IL-37, TGF-β, JNK/pSmad3L/c-Myc, pSmad3C/p21

## Abstract

IL-37 has been characterized as a fundamental inhibitor of innate immunity and a tumor suppressor in several cancers. However, the molecular mechanism of IL-37 in hepatocellular carcinoma (HCC) is largely unclear. In this study we found IL-37 expression was down-regulated in human HCC tissues and cell lines, and was negatively correlated with tumor size, vascular invasion, as well as overall-survial and disease-free survival (OS and DFS) of HCC. Multivariate Cox analysis revealed that IL-37 was an independent prognostic indicator for OS and DFS in HCC. Functional studies further showed that IL-37 overexpression significantly suppressed tumor growth by confining HCC to G2/M cell cycle arrest *in vitro* and *in vivo*. Mechanistically, we determined that IL-37 promoted Smad3 phospho-isoform signaling conversion from JNK/pSmad3L/c-Myc oncogenic signaling to pSmad3C/p21 tumor-suppressive signaling. Consistently, we detected a significant negative correlation between IL-37 expression and pSmad3L levels in a cohort of HCC biopsies; and the expression of pSmad3L predicted poorer outcome. These data highlight the importance of IL-37 in the cell proliferation and progression of HCC, and suggests that IL-37 may be a valuable biomarker for HCC prognosis.

## INTRODUCTION

Hepatocellular carcinoma (HCC) is the major type of primary liver cancer and is one of the most prevalent human malignancies globally [1, 2]. Although conventional clinicopathological characteristics, such as the alpha fetoprotein (AFP), hepatitis virus infection, tumor stage, histological grade, tumor size, microvascular invasion are generally regarded as prognostic factors for HCC patients, biomarkers are needed in clinical practice. Similarly, a large number of genetic variants and signaling pathways have been associated with HCC, but the contribution of these genes to the process of carcinogenesis remains unclear. Clear evidence has been shown that chronic inflammation plays a critical role in tumorigenesis; for example, production of tumor-promoting cytokines such as TGF-β, IL-17, IL-6, IL-8, TNF-α by immune/inflammatory cells that stimulate cell proliferation and survival is a major tumor-promoting mechanism [3–7]. However, the exact molecular mechanisms involved in inflammation-associated tumor are not completely understood.

Interleukin 37(IL-37), a recently identified member of the IL-1 family, was originally defined as *IL1F7* in 2010 [8]. The human IL-37 gene undergoes alternative splicing that results in five different isoforms (IL-1F7a through e) [9]. IL-37b has been suggested as a fundamental inhibitor of both innate and adaptive immunity. In a recent report, it was shown that Smad3 was required for anti-inflammatory effects of IL-37b, and either blocking Smad3 activation or Smad3 knockdown reduced the anti-inflammatory activity of IL-37b [10]. Interestingly, Smad3 mediates intracellular TGF-β signaling which plays an important role in cell proliferation, metastasis, cell survival, and epithelial-mesenchymal transition (EMT) [11]. As a transcription factor, Smad3 translocates from the cytoplasm to the nucleus, leading to regulated expression of its target genes [12]. TGF-β signaling during human hepatocellular carcinogenesis involves a shift in TGF-β function. The biological activities of TGF-β are initiated by the binding of the ligand to TGF-β receptors, which phosphorylate Smad proteins. TβRI activates Smad3 to create COOH-terminally phosphorylated Smad3 (pSmad3C), while pro-inflammatory cytokine-activated JNK phosphorylates Smad3 to create the linker phosphorylated Smad3 (pSmad3L) [13]. Previously, studies reported that TβRI/pSmad3C pathway inhibits growth of normal cells as a tumor suppressor, while JNK/pSmad3L-mediated signaling promotes tumor cell invasion as a tumor promoter during human hepatocarcinogenesis and ulcerative colitis-associated carcinogenesis [14, 15]. Moreover, Linker phosphorylation of Smad3 indirectly inhibits Smad3 C-terminal phosphorylation and subsequently suppresses pSmad3C signaling, The TβRI/pSmad3C and JNK/pSmad3L signals oppose each other, and the balance could shift from tumor suppression to carcinogenesis [16, 17].

Very recently, it has been demonstrated that IL-37 not only affects anti-inflammatory responses, but could also play a protective role in tumor progression. Gao et al. reported that the intratumoral injection of Ad-IL-37 resulted in significant growth suppression [18]. Moreover, Zhao et al. reported that the expression of IL-37 was decreased in tumor tissues of hepatocellular carcinoma patients, and and the expression level was negatively correlated with tumor size. High expression of IL-37 in HCC tumor tissues was associated with better overall survival (OS) and disease-free survival (DFS) [19]. In addition, IL-37 has been shown to suppress cell proliferation and invasion of human cervical cancer (CC) and Renal cell carcinoma (Rcc) through inhibiting signal transducer and activator of transcription 3 (STAT3) signaling [20, 21]. In 2016, Ge et al found that the expression of IL-37 was decreased in Non-small cell lung cancer (NSCLC) tissues and the antitumor activity of IL-37 was found by inhibition of angiogenesis *in vitro* and *in vivo* [22]. However, the biological functions and the exact molecular mechanisms of IL-37 in hepatocarcinogenesis remain largely unexplored.

In this study, we found lower expression of IL-37 in HCC tissues compared to adjacent non-cancerous tissues. Furthermore, IL-37 overexpression significantly suppressed HCC cells proliferation by confining HCC to G2/M cell cycle arrest *in vitro*. Moreover, IL-37 promoted Smad3 phospho-isoform signaling conversion from JNK/pSmad3L/c-Myc oncogenic signaling to pSmad3C/p21 tumor-suppressive signaling. Thus, the IL-37-pSmad3L axis may be a powerful predictor of poor prognosis in HCC.

## RESULTS

### IL-37 is poorly expressed in human HCCs and predicts poor prognosis

**Figure 1:**
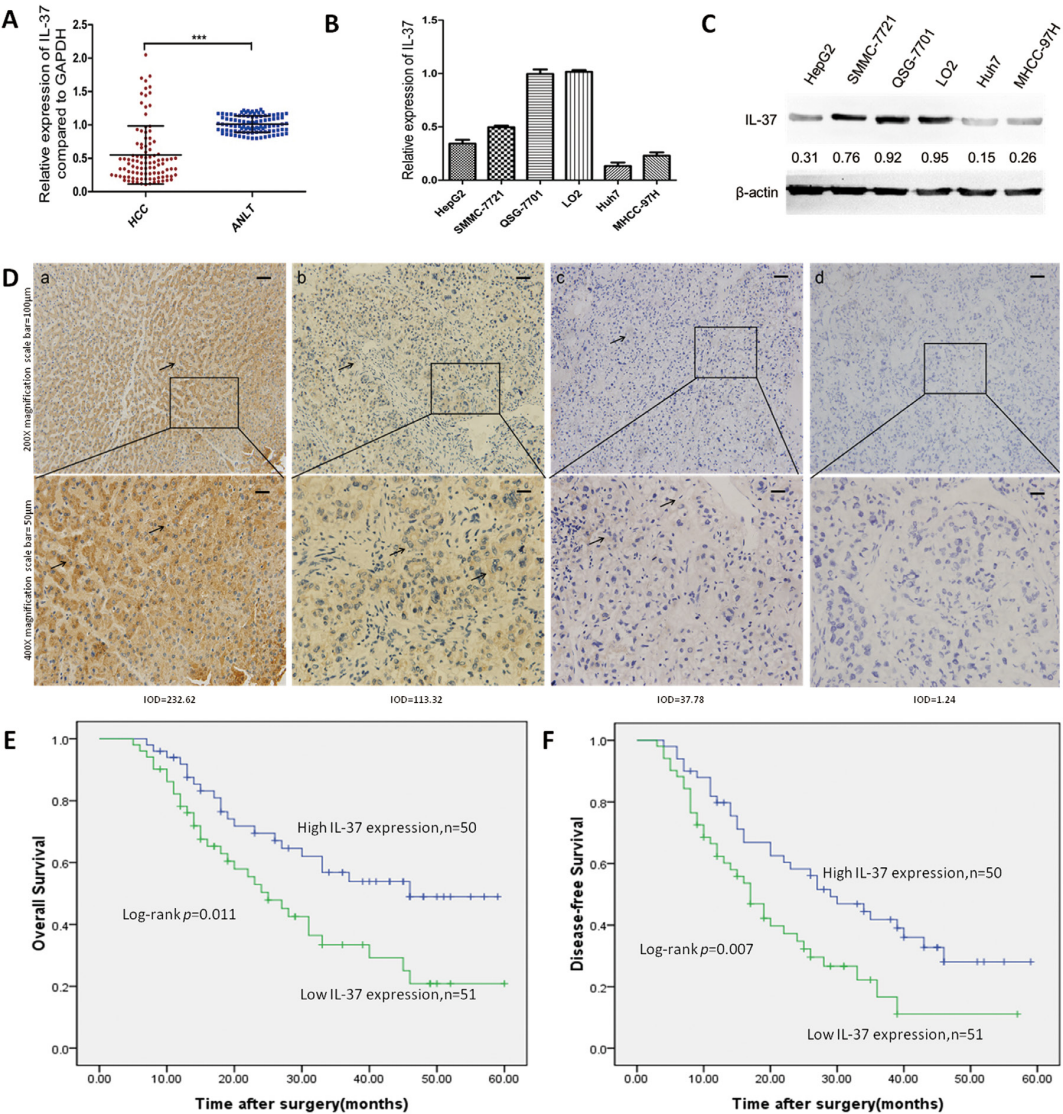
IL-37 is frequently down-regulated in human HCC and predicts a poor prognosis **A.** Expression of IL-37 in 101 pairs of HCC tissues and the corresponding ANLTs. Expression levels of IL-37 were normalized to the corresponding levels of GAPDH. Data were analyzed using a ΔΔCt method approach and expressed as log2 fold change (ΔΔCt [HCC/ANLT]). ***P < 0.001. **B.** Relative IL-37 mRNA levels in nonneoplastic liver cell line(QSG-7701 and LO2) and HCC cell lines (HepG2, SMMC-7721, Huh7, and MHCC97H) by real-time PCR. Expression levels of IL-37 were normalized to the corresponding levels of GAPDH. Each sample was analyzed in triplicate and values are expressed as levels (mean ±SD) relative to those in L02 cells. **C.** Expression of IL-37 in HCC cell lines and nonneoplastic liver cell line(QSG-7701 and LO2) detected by Western blotting, β-actin was used as internal reference for IL-37 protein. Expression levels of IL-37 were normalized to the corresponding levels of β-actin. **D.** IL-37 protein expression in primary hepatocellular carcinoma surgical specimens as shown by immunohistochemical detection:a) IL-37 expression in distant normal liver tissues. (b) and (c) IL-37 positively staining in tumor cases. (d) IL-37 negative staining in tumor case.IOD^a^=232.62, IOD^b^=113.32, IOD^c^=37.78, IOD^d^=1.24. **E-F.** Survival analysis on the basis of IL-37 expression in HCCs. According to the IHC data, the expression of IL-37 was classified into low expression group(n=51) and high expression group(n=50). Survival curves were constructed using the Kaplan-Meier method and evaluated using the log-rank test.

To investigate the clinical significance of IL-37 in HCC, we first detected its expression in 101 pairs of HCC tissues and their non-cancerous counterparts by qRT-PCR. Compared with the adjacent non-tumor liver tissues (ANLTs), significantly lower expression of IL-37 mRNA was observed in 85.15% (86/101) of HCC samples (Figure 1A), suggesting that reduction of IL-37 was a frequent event in human HCC. We also measured the expression of IL-37 in both human HCC cell lines and nonneoplastic liver cell line(QSG-7701 and LO2) by qRT-PCR and Western blotting. We found that mRNA and protein expression of IL-37 was lower in the human HCC cell lines compared with human liver cells (Figure 1B and 1C). To further confirm this result, IHC analysis was performed in HCC tissues and ANLTs. Consistent with the qRT-PCR findings, IL-37 had the higher expression in ANLTs, and decreased expression in HCC tissues. Positive staining of IL-37 was mainly detected in the ANLTs, and was located in the cytoplasm of the hepatocytes (Figure 1D). In HCC tumor tissues, there was variation in the level of IL-37 expression (Figure 1D). Based on the IHC results, All 101 patients with HCC were divided into two groups: the high-expression (n=50) and low-expression group (n=51) to investigate the association between the IL-37 expression and the clinical features in HCC patients. We determined that IL-37 expression was significantly associated with tumor size (*P*=0.046), tumor Barcelona Clinic Liver Cancer (BCLC) staging (*P*=0.030) and microvascular invasion (*P*=0.046) (Table 1).

**Table 1:** Correlations between IL-37 Expression level and clinicopathological variables of 101 cases of HCC

Clinicopathological variables	Number of each group	IL-37 expression		*p* value
		high	low	
All cases	101	50	51	
Age (years)				0.549
<50	44	20	24	
≥50	57	30	27	
Gender				0.842
Male	55	28	27	
Female	46	22	24	
Tumor size(cm)				**0.046**
<5	44	27	17	
≥5	57	23	34	
Histological differentiation				0.112
Well	39	18	21	
Moderate	30	19	11	
Poor	32	13	19	
Liver cirrhosis				0.216
Absence	36	21	15	
Presence	65	29	36	
Serum AFP (ug/ml)				0.836
≤400	35	18	17	
>400	66	32	34	
BCLC stage				**0.030**
A	18	13	5	
B	36	19	17	
C	47	18	29	
HBV				1.000
Negative	11	5	6	
Positive	90	45	45	
Microvascular invasion				**0.046**
Yes	47	18	29	
No	54	32	22	

AFP, α-fetoprotein; BCLC stage, Barcelona Clinic Liver Cancer stage; HBV, hepatitis B virus;

To assess the feasibility of IL-37 expression in HCC prognosis, Cox proportional hazards regression model was performed. On univariate survival analysis revealed histologic grade (*P*=0.009), tumor staging (BCLC) (*P*=0.033), microvascular invasion (*P*=0.030), and IL-37 expression (*P*=0.013) as significant variables for OS. Next, Multivariate Cox regression analyses showed that OS was significantly dependent on histologic grade (*P*=0.017) and IL-37 expression levels (*P*=0.050; Table 2). Furthermore, HCC patients with low IL-37 expression had much poorer OS (median survival time, 25.0 *versus* 46.0 months, *P*=0.011; Figure 1E) than patients with high IL-37 expression. Univariate analysis indicated that tumor size (*P*=0.023), histologic grade (*P*=0.005), tumor staging (BCLC) (*P*=0.017), microvascular invasion (*P*=0.009) and IL-37 expression levels (*P*=0.009) reached significance for DFS. Next, multivariate survival analysis on all parameters was performed. We found that DFS was significantly dependent on histologic grade (*P*=0.010), microvascular invasion (*P*=0.046), and IL-37 expression levels (*P*=0.045) (Table 3). Similarly, HCC patients with high IL-37 expression had longer DFS (median survival time, 29.0 *versus* 17.0 months, *P*= 0.007; Figure 1F). These results suggested that reduced IL-37 expression was a frequent event in human HCC and might be involved in liver carcinogenesis.

**Table 2:** Univariate and multivariate analysis of overall survival (OS) in hepatocellular carcinoma

Variables	n	Univariate Analysis		Multivariate Analysis	
		HR (95% CI)	*P*	HR (95% CI)	*P*
Age (years)					
<50	44	0.791(0.455-1.374)	0.405		
≥50	57				
Gender					
Female	46	0.726(0.418-1.259)	0.254		
Male	55				
Tumor size(cm)					
<5	44	0.576(0.330-1.006)	0.053		
≥5	57				
Histologic grade					
Poor	39	2.558(1.267-5.164)	**0.009**	2.377(1.164-4.854)	**0.017**
Moderate	32				
Well	30				
Liver cirrhosis					
Absence	36	0.792(0.440-1.425)	0.437		
Presence	65				
Serum AFP (ng/ml)					
≤400	35	0.914(0.524-1.594)	0.752		
>400	66				
BCLC stage					
A	18	0.372(0.150-0.923)	**0.033**	0.480(0.169-1.364)	0.168
B	36				
C	47				
HBV					
Negative	11	0.377(0.117-1.212)	0.102		
Positive	90				
Microvascular invasion					
NO	54	0.546(0.316-0.942)	**0.030**	0.617(0.338-1.124)	0.115
Yes	47				
IL-37 expression					
High	51	0.496(0.285-0.863)	**0.013**	0.555(0.308-0.999)	**0.050**
Low	50				

AFP, α-fetoprotein;BCLC stage, Barcelona Clinic Liver Cancer stage;HBV, hepatitis B virus;

**Table 3:** Univariate and multivariate analysis of Disease-free survival (DFS) in hepatocellular carcinoma

Variables	n	Univariate Analysis		Multivariate Analysis	
		HR (95% CI)	*P*	HR (95% CI)	*P*
Age (years)					
<50	44	0.890(0.549-1.444)	0.638		
≥50	57				
Gender					
Female	46	0.697(0.428-1.134)	0.228		
Male	55				
Tumor size(cm)					
<5	44	0.560(0.340-0.923)	**0.023**	0.620(0.363-1.059)	0.080
≥5	57				
Histologic grade					
Poor	39	2.395(1.293-4.437)	**0.005**	2.263(1.212-4.225)	**0.010**
Moderate	32				
Well	30				
Liver cirrhosis					
Absence	36	1.047(0.639-1.715)	0.855		
Presence	65				
Serum AFP (ng/ml)					
≤400	35	0.765(0.462-1.267)	0.299		
>400	66				
Tumor staging(BCLC)					
A	18	0.356(0.153-0.829)	**0.017**	0.869(0.342-2.209)	0.769
B	36				
C	47				
HBV					
Negative	11	0.623(0.269-1.445)	0.270		
Positive	90				
Microvascular invasion					
NO	54	0.527(0.325-0.853)	**0.009**	0.582(0.342-0.991)	**0.046**
Yes	47				
IL-37 expression					
High	51	0.516(0.315-0.845)	**0.009**	0.531(0.285-0.987)	**0.045**
Low	50				

AFP, α-fetoprotein;BCLC stage, Barcelona Clinic Liver Cancer stage;HBV, hepatitis B virus;

### Overexpression of IL-37b significantly suppresses HCC growth and proliferation by inducing cell growth arrest in G2 phase *in vitro*

**Figure 2:**
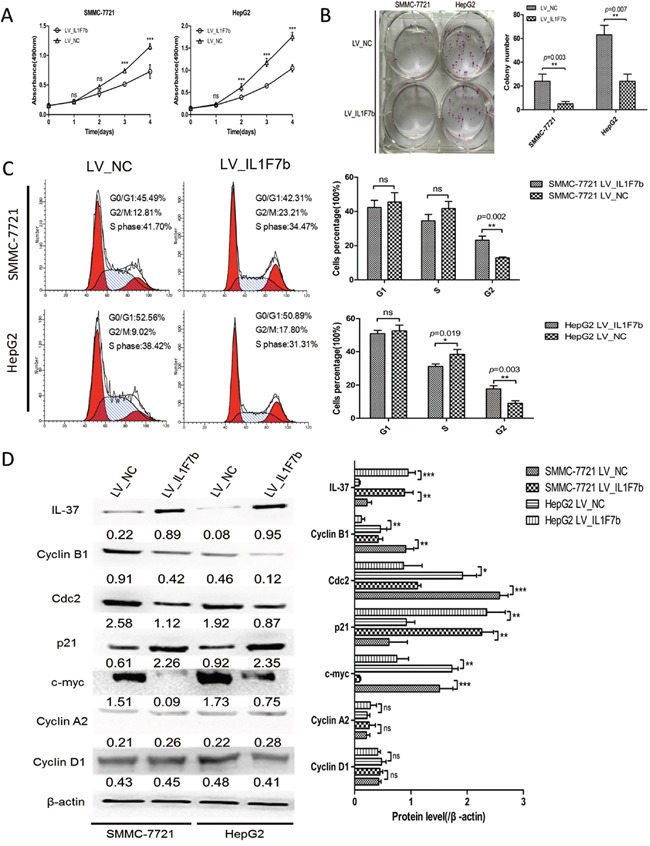
IL-37b inhibits HCC cell growth and colony formation *in vitro* **A.** The growth of HCC SMMC-7721 and HepG2 cells with IL-37b overexpression or vector control was determined as described in Materials and Methods. ***P < 0.001. **B.** The colony formation assay was performed as described in Materials and Methods. The number of colonies was counted and compared. **C.** The cell cycle distribution of SMMC-7721 and HepG2 cells infected with LV_*IL1F7b* or LV_NC were analyzed as described in Materials and Methods. **D.** The protein expression levels of IL-37, cdc2, p21, c-myc, cyclin B1, cyclin A2, cyclin D1 were estimated in SMMC-7721 and HepG2 cells with IL-37b overexpression or vector control by Western blot analysis. β-actin was used as an internal control. The data shown are representative of three independent experiments with similar results. Data are expressed as mean±SD; Student's *t* test; ^ns^*p*> 0.05; *p< 0.05, **p< 0.01, and ***p< 0.001.

To examine the potential role of IL-37b in biological functions, we initially evaluated the effect of IL-37b on the growth and clonogenicity of cancer cells *in vitro*. We up-regulated IL-37b expression by infecting LV_*IL1F7b* or LV_NC in highly proliferous SMMC-7721 and HepG2 cells. We confirmed the up-regulation of IL-37b in those cell lines by Western blot analysis (Figure 2D). We observed that up-regulation of IL-37b protein resulted a significant suppression in cell proliferation (Figure 2A), and colony formation (Figure 2B). To investigate the mechanisms of IL-37b suppression in HCC cell proliferation, we examined the effects of IL-37b on apoptosis and cell cycle by flow cytometry. We found that the decrease in the viable cell numbers could not be attributed to the induction of apoptosis (Supplementary Figure S1). But compared with vector control cells, overexpression of IL-37b significantly increased the proportion of cells in G2/M phase from 12.81%±0.57% to 23.21%±2.39% in SMMC-7721 cells, and from 9.02%±1.49% to 17.80%±1.87% in HepG2 cells (Figure 2C). Furthermore, overexpression of IL-37b inhibited the expression of G2/M phase checkpoint proteins cyclin B1, cdc2 and c-myc, and increased the expression of p21; while there were no significant changes in the protein level of other cyclins (cyclin A2 and cyclin D1; Figure 2D). These results revealed that enforced expression of IL-37b caused a marked accumulation of G2 population in different cell lines compared to controls.

### Knockdown of IL-37 significantly promotes HCC cells growth and proliferation *in vitro*

**Figure 3:**
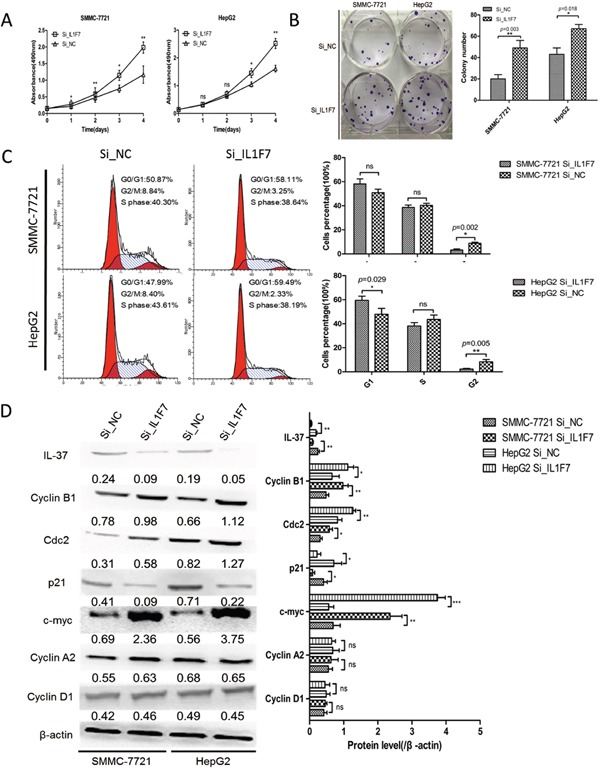
IL-37 knockdown facilitated cell proliferation and colony formation of HCC cell *in vitro* SMMC-7721 and HepG2 cells were transfected with negative control siRNA (Si_NC) or IL-37-specific siRNA (Si_IL1F7). **A.** Cell growth assays were performed using a CCK-8 kit at the indicated times. Absorbance values were used to indicate the changes in proliferative activity. **B.** The colony formation assay was performed as described in Materials and Methods. The number of colonies was counted and compared. **C.** Flow cytometric analysis of the cell cycle in SMMC-7721 and HepG2 cells transfected with Si_NC or Si_IL1F7. Experiments were repeated three times with similar trends. **D.** Representative Western blots of IL-37, cdc2, p21, c-myc, cyclin B1, cyclin A2, and cyclin D1 proteins in the indicated SMMC-7721 and HepG2 cells. The data shown are representative of three independent experiments with similar results. Data are expressed as mean±SD; Student's *t* test; ^ns^*p*> 0.05; *p< 0.05, **p< 0.01, and ***p< 0.001.

Abrogation of IL-37 expression *via* siRNA was investigated to assess the potential consequences of IL-37 silencing. Down-regulation of IL-37 was confirmed by Western blotting (Figure 3D). The results showed that the growth of Si_*IL1F7* group was significantly faster than that of Si_NC group in both SMMC-7721 and HepG2 cells (Figure 3A). As shown in Figure 3B, the colony numbers were 20 ± 4 vs. 49 ± 7 in SMMC-7721 cells (*P*=0.003) and 43 ± 6 vs. 67 ± 4 in HepG2 cells (*P*=0.018) before and after IL-37 knockdown. These results suggested that silencing of IL-37 promoted cells proliferation. To examine the way in which this occurred through cell cycle alterations, flow cytometry technique was employed. The cell phase distribution of Si_*IL1F7* and Si_NC SMMC-7721 cells was as follows: G1 phase: 58.11 ± 4.18% vs. 50.87 ±2.89%; S phase: 38.64 ±1.92% vs. 40.30 ±1.72%; G2/M phase: 3.25 ±0.91% vs. 8.84 ±0.86% (*P*=0.002). The cell phase distribution of Si_*IL1F7* and Si_NC HepG2 cells was as follows: G1 phase: 59.49 ± 3.47% vs. 47.99 ± 4.82% (*P*=0.029); S phase: 38.19 ± 2.78% vs. 43.61 ± 3.67%; G2/M phase: 2.33 ±0.51% vs. 8.40 ±1.82% (*P*=0.005). For both cell lines, G2/M phase was significantly decreased in Si_*IL1F7* group cells (Figure 3C). To further validate the effect of IL-37 depletion on the cell cycle, the expression of G2/M phase checkpoint proteins cyclin B1, cdc2 and c-myc, p21 was examined by Western blot (Figure 3D). Cyclin B1, cdc2 and c-myc were apparently increased in Si_*IL1F7* group cells and decreased the expression of p21;while there were also no significant changes in the protein level of other cyclins (cyclin A2 and cyclin D1). Taken together, these data indicated that IL-37 suppressed HCC cells growth and proliferation in by regulating the G2/M transition of the cells.

### IL-37b inhibits HCC growth through converting Smad3 phospho-isoform

**Figure 4:**
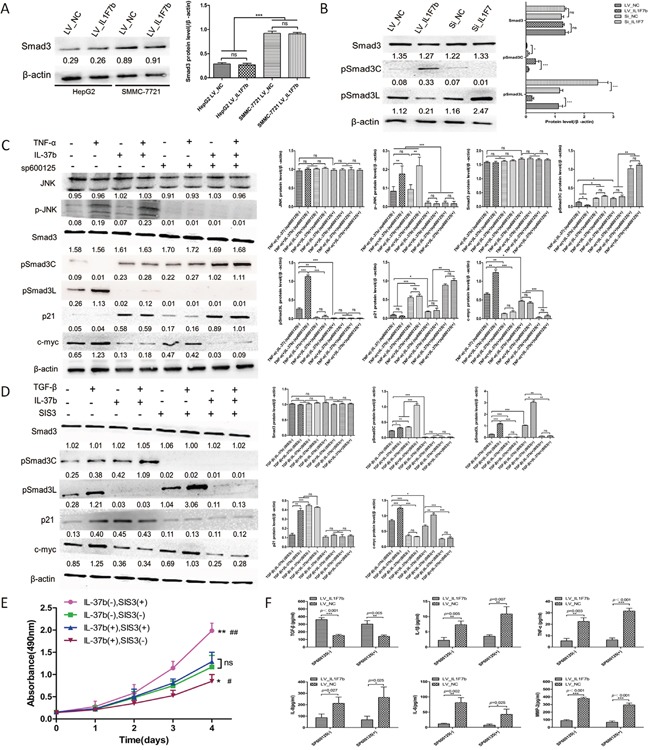
IL-37b converts hepatocytic pSmad from pSmad3C/p21 tumor-suppressive signaling to JNK/pSmad3L/c-Myc oncogenic signaling **A.** Western blot results of Smad3 proteins in SMMC-7721 and HepG2 cells infected with LV_*IL1F7b* or LV_NC. **B.** Western blot results of endogenous pSmad3C and pSmad3L proteins in IL-37b-overexpression or IL-37-knockdown SMMC-7721 cells. **C.** IL-37b inhibited TNF-α mediated activation of the pSmad3L/c-myc pathway but potentiated pSmad3C/ p21 expression in SMMC-7721 cells. Serum-starved LV_NC and LV_*IL1F7b*-infected SMMC-7721 cells were incubated for 8 h in the absence or presence of 10 μM SP600125 and were then treated for 30 mins with 400 pM TNF-α. Expression of endogenous Smad3 phosphorylation, p21, and c-myc was analyzed in Western blot using specific Abs. **D.** TGF-β1-dependent Smad3 phosphorylation inhibitor, SIS3, inhibited IL-37, as well as TGF-β-mediated pSmad3C/p21 signal activation. Serum-starved LV_NC and LV_*IL1F7b*-infected SMMC-7721 cells were incubated for 8 h in the absence or presence of 2 μM SIS3 and were then treated for 30 mins with 20 pM TGF-β1. Expression of endogenous Smad3 phosphorylation, p21, and c-myc was analyzed in Western blot using specific Abs. **E.** LV_NC and LV_*IL1F7b*-infected SMMC-7721 cells were incubated for 8 h in the absence or presence of 2 μM SIS3, Cell growth assays were performed using a CCK-8 kit at the indicated times. Absorbance values were used to indicate the changes in proliferative activity.*P<0.05;**P<0.01 compared with IL-37b(-)SIS3(-),#P<0.05;##P<0.01 compared with IL-37b(+)SIS3(+). **F.** LV_NC and LV_*IL1F7b*-infected SMMC-7721 cells were cultured for 8 h in the absence or presence of 10 μM SP600125, and the pro-inflammatory cytokines in culture supernatants were measured by ELISA. The data shown are representative of three independent experiments with similar results. Data are expressed as mean±SD;.Student's *t* test; ^ns^*p*> 0.05; *p< 0.05, **p< 0.01, and ***p< 0.001. LSD and S–N–K Test; ns*p*> 0.05; *p< 0.05, **p< 0.01, and ***p< 0.001.

Previous studies have suggested that IL-37b and Smad3 form a functional complex and Smad3 is required for IL-37b activity in anti-inflammation. Because TGF-β signaling occurs during human hepatocellular carcinogenesis and involves a shift in TGF-β/Smad3 function [26], we speculated that the tumor-suppressive function of IL-37b might be implicated in TGF-β signaling. To examine this hypothesis and further determine whether IL-37b requires Smad3 to carry out its anti-tumor activity, we first evaluated the Smad3 protein level in SMMC-7721 and HepG2 cells. The results showed that the Smad3 protein level of SMMC-7721 cells was higher than HepG2 cells (Figure 4A). So we choosed the SMMC-7721 cell line to investigate the potential molecular mechanisms. We next investigate the effect of IL-37b on the phosphorylation of Smad3 in SMMC-7721 cells. As expected, IL-37b notably decreased expression of pSmad3L and increased pSmad3C expression, the effects which were reversed upon IL-37 inhibition by siRNA (Figure 4B). It is known that TGF-β can induce Smad3 phosphorylation at its C-terminal region by TGF-β type I receptor (TβRI) and TNF-α-dependent phosphorylation occurs on two serine residues within the linker region but not within the C-terminal region by c-Jun N-terminal kinase (JNK) [16, 17, 27]. To investigate the molecular regulation mechanism of domain-specific phosphorylation in Smad3 signaling by IL-37b, we first treated the aforementioned LV_NC and LV_*IL1F7b* cells with TNF-α to determine the regulative effects of IL-37b on TNF-α/JNK-dependent phosphorylation of Smad3. TNF-α treatment resulted in the activation of pSmad3L/c-myc and inactivation of pSmad3C/p21 in LV_NC cells (Figure 4C, lane 1 *versus* lane 2). However, TNF-α-mediated activation of pSmad3L/c-myc signal was dramatically attenuated in the presence of IL-37b, and in the meantime, pSmad3C and p21 were upregulated (Figure 4C, lane 2 *versus* lane 4). In addition, IL-37b did not have a significant effect on phosphorylation of JNK (Figure 4C, lane 1 *versus* lane 3) which indicated that the target of IL-37b was downstream of JNK. Since phosphorylation of Smad3 at the linker region inhibits C-terminal phosphorylation induced by TβRI [17], we investigated whether JNK activity affected these effects. Pre-treatment of hepatocytes with a JNK inhibitor (SP600125) severely abolished the subsequent increase in pSmad3L and c-myc expression (Figure 4C, lane 1 *versus* lane 5). Theoretically, the pSmad3C signal should not be effected in SP600125 pretrentment LV_*IL1F7b* cells. But as shown in Figure 4C, We found that pSmad3C and p21 were still up-regulated in LV_*IL1F7b* cells with SP600125 pretrentment (Figure 4C, lane 5,6 *versus* lane 7,8). We therefore hypothesized that this observation might be attributed to IL-37b-mediated secretion of TGF-β that subsequently activates pSmad3C/p21 signal. To test this hypothesis, we performed ELISA, and as shown in Figure 4F, compared with the negative control, TGF-β was markedly up-regulated while pro-inflammatory cytokines IL-6, IL-8, IL-1β, TNF-α and MMP2 levels were dramatically down-regulated in the supernatants of both SP600125-pre-treated and non-pre-treated LV_*IL1F7b* cells. As the direct target of IL-37b was still unclear, we further investigated the regulative effects of IL-37b on TGF-β/pSmad3C/p21 signaling. The expression of pSmad3C and p21 was markedly up-regulated by TGF-β stimulation and/or IL-37b overexpression; however, the expression of pSmad3C and the up-regulation of p21 was dramatically abolished in the presence of SIS3 which selectively inhibits TGF-β1-dependent Smad3 phosphorylation (Figure 4D). Importantly, the pSmad3L/c-myc signal was still attenuated or completely abolished upon TGF-β stimulation and/or SIS3 pre-treatment in LV_*IL1F7b* cells. (Figure 4D). This finding demonstrated that IL-37b did not affect pSmad3C/p21 signaling directly. To further elucidate the tumor-suppressive functions of IL-37b whether through TGF-β signaling conversion. We tested whether SIS3 could affect growth inhibition function of IL-37b by using cck-8 assay. As shown in Figure 4E, the inhibitory effect of IL-37b on HCC cell proliferation was abrogated by SIS3 pre-treatment. Taken together, these data indicated that IL-37b may be a direct inhibitor of JNK/pSmad3L/c-myc signaling and promotes Smad3 phospho-isoform signaling shift from JNK/pSmad3L/c-myc oncogenic signaling to pSmad3C/p21 tumor-suppressive signaling.

### IL-37b suppress HCC progression *in vivo*

**Figure 5:**
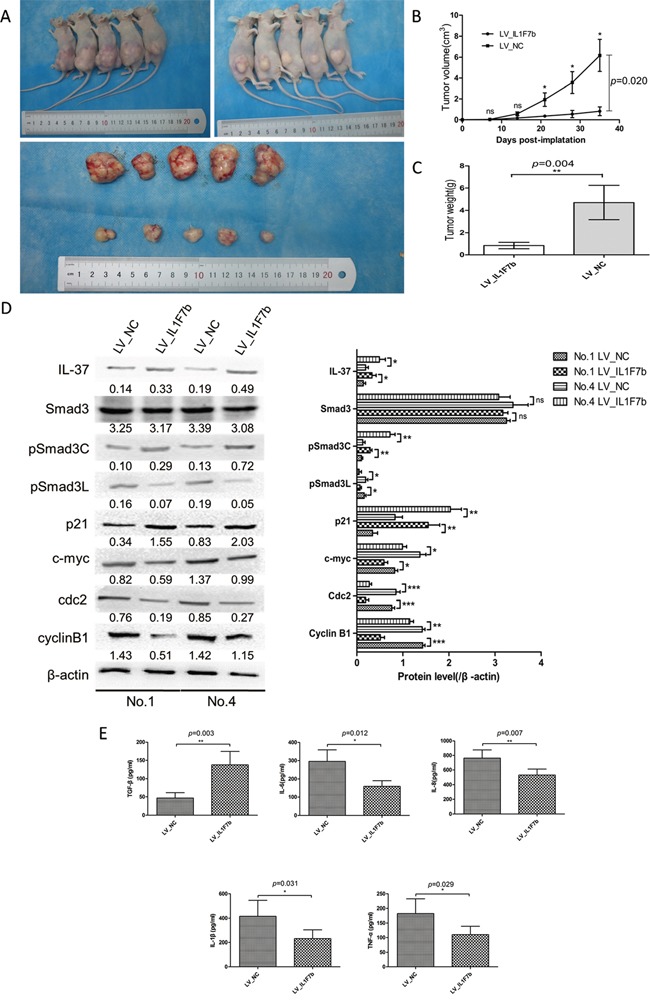
IL-37b suppresses HCC progression in nude mice **A-B.** The tumor growth rate of LV_*IL1F7b* group was drastically decreased relative to LV_NC [mean tumor volume (NC *vs*. IL-37), 6.170±1.530 *vs*. 0.818±0.422 cm3, *p*<0.001. **C.** mean tumor weight (NC *vs*. *IL1F7b*, 4.700±1.544 *vs*. 0.840±0.288 g, *p*=0.004, respectively). **D.** Western blot analysis of representative excised tumors in A. **E.** Overexpression of IL-37 markedly down-regulated production of IL-6, IL-8, IL-1β, and TNF-α in mice serum and up-regulated production of TGF-β as measured by ELISA. Data are expressed as mean± SD; Student's t test; **p< 0.01, and ***p < 0.001.

To confirm the *in vitro* data, we further examined the effect of IL-37b on HCC tumorigenicity by establishing a xenotransplantation of tumor grafts in nude mice. LV_*IL1F7b* or LV_NC SMMC-7721 cells were injected subcutaneously into the anterior huckle of the nude mice and observed for tumor growth. Compared with LV_NC group, LV_*IL1F7b* group led to a decreased mean size of tumor volume (NC *vs. IL1F7b*, 6.170±1.530 *vs.* 0.818±0.422 cm3, *P*<0.001, Figure 5A, 5B) and mean tumor weight (NC *vs*. *IL1F7b*, 4.700±1.544 *vs.* 0.840±0.288 g, *P*=0.004, Figure 5C), respectively. As expected, the tumors in mice inoculated with LV_*IL1F7b* cells showed reduced levels of pSmad3L, Cyclin B1, cdc2 and c-myc, while the expression of pSmad3C and p21 was upregulated (Figure 5D). LV_*IL1F7b* group also increased the serum production of TGF-β, while the production of IL-6, IL-8, IL-1β, TNF-α were markedly decreased (Figure 5E).

### Expression of pSmad3L and the correlation between IL-37 and pSmad3L in HCC samples

**Figure 6:**
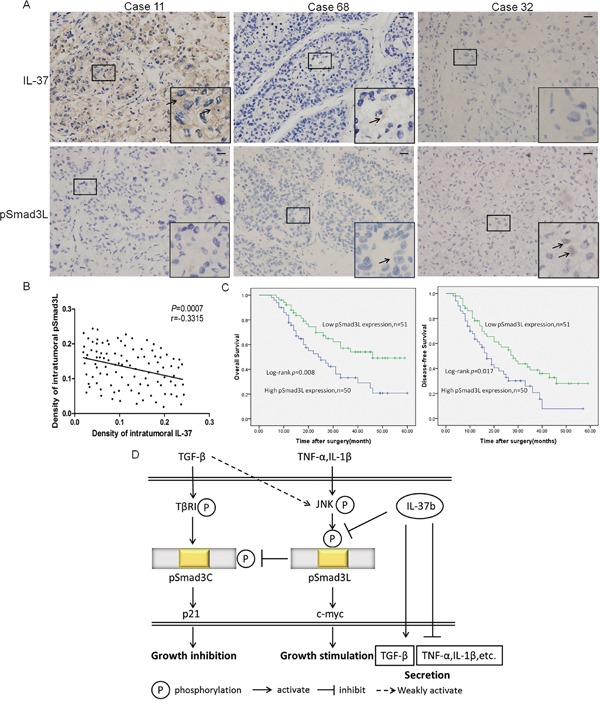
Expression of pSmad3L and its correlation with IL-37 in HCC patients **A.** Representative immunostaining for pSmad3L and IL-37 is shown for three patient samples(400×magnification; Scale bar=50 μm). **B.** Correlation between pSmad3L expression and IL-37 level was examined in tumor tissues derived from 101 patients, r = -0.3315, *p*=0.0007. **C.** Survival relevance analysis of pSmad3L expression in HCCs. According to the IHC data, the expression of pSmad3L was classified into low expression (n=51) and high expression (n=50). Survival curves were constructed using the Kaplan-Meier method and evaluated using the log-rank test. **D.** A schematic model depicting the hypothesized molecular mechanism: IL-37b targets pSmad3L to suppress the HCC growth by shifting Smad3 phospho-isoform signaling from JNK/pSmad3L/c-Myc oncogenic signaling to pSmad3C/p21 tumor-suppressive signaling.

Since IL-37b notably down regulated expression of pSmad3L, we speculated that IL-37 protein level was negatively correlated with pSmad3L protein level in human HCC tissues. To test this hypothesis, pSmad3L expression was evaluated by immunohistochemistry in forementioned 101 HCC tissues. Positive staining of pSmad3L was mainly detected in the tumor tissues, and was located in the nucleus of the tumor cells (Figure 6A). As expected, IHC staining revealed a negative correlation of IL-37 expression with pSmad3L levels (Figure 6B). Based on the IHC results, All 101 patients with HCC were divided into two groups: the high-expression (n=50) and low-expression group (n=51), Kaplan-Meier survival analysis of pSmad3L expression revealed that HCC patients with high pSmad3L expression had poorer OS (*P*=0.008) and progression-free survival (PFS) (*P*=0.017) than those with low pSmad3L expression, indicating that pSmad3L predicts poorer clinical outcome (Figure 6C). In conclusion, evaluation of both IL-37 expression and pSmad3L signal may be a powerful predictor of poor prognosis in HCC.

## DISCUSSION

Efforts to elucidate the molecular mechanism underlying HCC tumorigenicity are warranted in order to identify biomarkers for prediction and intervention [28]. Recently, it has been revealed that altered expression of IL-37 contribute to the initiation and progression of cancer [29]. Wang et al. [20] found that IL-37 acts as a tumor suppressor by controlling STAT3 activity in CC. They validated that impaired STAT3 function reduced the ability of IL-37 to suppress the cell proliferation and invasion. Zhao et al. [19] reported that IL-37 expression is lower in primary HCC tumor tissues and is associated with tumor progress and poor prognosis.IL-37 mediated anti-tumor immune responses through recruiting CD57+ NK cells to tumor microenvironment in HCC, but the detailed mechanism of IL-37's effect on hepatocarcinogenesis is still unclear. In the present study, Similar to Zhao's findings, We verified that IL-37 was frequently down-regulated in both HCC tissues and liver cancer cell lines. But we further found that intratumoral IL-37 expression was negative correlated with BCLC stage and microvascular invasion indicating that IL-37 expression might inhibit HCC tumorigenicity. The discrepancy may be due to different backgrounds of specimens used and the criteria of tumor staging in primary HCC. Kaplan-Meier analysis showed that HCC patients with low IL-37 expression in general had worse prognosis than those with higher IL-37 expression and cox multivariate analyses showed that IL-37 is an independent risk factor indicating that IL-37 is an attractive candidate gene for risk prognostication and therapy of HCC.

Given that IL-37 was down-regulated in HCC tissues and cell lines, we speculated that regulation of IL-37 might influence the malignant phenotypes of HCC cells. The results derived from *in vitro* cell proliferation, colony formation and *in vivo* tumor progression assays confirmed that overexpression of IL-37b suppresses the potency of HCC cell growth and proliferation, which were reversed upon IL-37 knockdown. We further demonstrated that IL-37b suppressed cell growth by promoting activation of cell cycle arrest, which was accompanied by changes in the expression of G2/M checkpoint proteins and c-myc. The cell cycle is regulated by cyclin and cyclin-dependent kinases. Cyclin B1 is a key molecule for G2/M phase transition of the cell cycle and is required for initiation of mitosis [30]. Because mitosis is directly controlled by cyclin B1, Overexpression of cyclin B1 results in uncontrolled cell growth and may promote tumorigenesis. This might be one of the mechanisms in genetic instability and carcinogenesis [31]. In addition to cell cycle arrest, IL-37b downregulated expression of the oncogene c-myc, which might be another molecular mechanism through which IL-37 suppresses HCC cell growth. Therefore, reduced expression of IL-37 induces cell cycle arrest, in turn inhibits cell proliferation and suppresses the development of HCC.

TGF-β signaling during human hepatocellular carcinogenesis involves a shift in TGF-β function. The biological activities of TGF-β are initiated by the binding of the ligand to TGF-β receptors, which phosphorylate Smad proteins. TβRI activates Smad3 to create COOH-terminally phosphorylated Smad3 (pSmad3C), while pro-inflammatory cytokine-activated JNK phosphorylates Smad3 to create the linker phosphorylated Smad3 (pSmad3L) [13]. Previously, studies reported that TβRI/pSmad3C pathway inhibits growth of normal cells as a tumor suppressor, while JNK/pSmad3L-mediated signaling promotes tumor cell invasion as a tumor promoter during human hepatocarcinogenesis and ulcerative colitis-associated carcinogenesis [14, 15]. Interestingly, a recent study has revealed that IL-37b potentially binds to both non-phosphorylated Smad3 and pSmad3C, silences endogenous Smad3, or blocks pSmad3C signal significantly to reduce the anti-inflammatory properties of IL-37b [10]. Therefore, the anti-carcinogenic capabilities of IL-37b might involve Smad3 phospho-isoform signaling. In the present study, we found that IL-37b was indeed involved in TGF-β function conversion. Our *in vitro* and *in vivo* studies suggested that IL-37b could suppress the JNK/pSmad3L signal and promote the pSmad3C/p21 signal. However, the direct target of IL-37b was still unclear, If the direct target of IL-37b is pSmad3C/p21 signal, then pSmad3C/p21 signal of IL-37b-overexpression cells should be activated and JNK/pSmad3L/c-Myc signal should not be inhibited upon SIS3 pre-treatment. However, we found that IL-37b had no such effect on pSmad3C/p21 signal activation upon SIS3 pre-treatment. Therefore, the direct target of IL-37b could be JNK/pSmad3L/c-Myc signal. To explain this observation, we speculate that the phosphorylation sites of pSmad3L may be shielded by the binding domain of IL-37b-Smad3 complex. We will analyze the potential IL-37b-Smad3 interactions by using co-immunoprecipitation (co-IP) and *in vitro* pull-down assays in future studies. Several pro-inflammatory cytokines such as IL-6, IL-8, IL-1β, TNF-α can be activated by IL-37 [32–34]. And consistent with those results, we found that IL-37b markedly promoted the secretion of TGF-β and while dramatically inhibiting the secretion of IL-6, IL-8, IL-1β, TNF-α and MMP2, suggesting that IL-37b establishes a positive feedback loop to regulate TGF-β/Smad3 signaling by promoting the secretion of tumor-suppressive cytokines and inhibiting the secretion of the oncogenic cytokines to achieve HCC tumor-suppression (Figure 6D). Additionally, we examined whether pSmad3L signal involved in IL-37b-mediated attenuation of oncogenic cytokines. Our data demonstrates that JNK inhibitor sp600125 does not reverse the IL-37b-mediated attenuation of oncogenic cytokines secretion (Figure 4F), indicating that IL-37b-induced attenuation of oncogenic cytokines secretion is not due to inactivation of pSmad3L signal in this study.

IL-37 has been shown to suppress cell proliferation and invasion of human cervical cancer (CC) and Renal cell carcinoma (Rcc) through inhibiting STAT3 signaling [18]. STAT3 is well demonstrated to play a crucial role in tumorigenesis and cancer-related inflammation [35]. Persistent activation of STAT3 signaling is involved in promoting tumor cell proliferation, metastasis, invasion, angiogenesis and immunosuppression [36, 37]. There are several earlier studies, albeit conflicting, reporting crosstalks between TGF-β and STAT3 signaling pathways. For instance, TGF-β negatively inhibits IL-6-mediated STAT3 activation and affects its target gene expression [38]. Conversely, STAT3 directly interacts with Smad3 *in vivo* and *in vitro*, resulting in attenuation of the Smad3-Smad4 complex formation and suppression of DNA-binding ability of Smad3, but did not obviously influence TGF-β-induced phosphorylation of endogenous Smad3(s425) [39]. In the present study, we did not investigate the interplays between STAT3 and TGF-β signaling pathways upon IL-37b treatment. But based on our current data and existing studies, It is not difficult to infer that STAT3 signaling and TGF-β/pSmad3C signaling are both regulated by IL-37b.Up-regulation of IL-37b may therefore contribute to the suppressive effects of STAT3 signaling pathway and could result in a conversion of Smad3 phospho-isoform signaling from JNK/pSmad3L/c-Myc oncogenic signaling to pSmad3C/p21 tumor-suppressive signaling. These two effects might play a independent and synergistical role in the anti-tumor process of IL-37b.However, is there a crosstalk between STAT3 and pSmad3L? Further studies are being planned to investigate. Taken together, these data suggested that IL-37b could directly suppress pSmad3L and promote the up-regulation of pSmad3C, further regulating downstream genes which modulate tumorigenicity, we would like to provide a gene regulatory network (Figure 6D).

Cancer and Chronic inflammation are two reciprocally regulated events. Tumor cells can usually generate a regional inflammatory environment, and conversely, inflammatory environment will further promote malignant transformation [40]. IL-37 is recognized as a major mediator in either immunoregulation or tumorigenesis. Previous studies have found that IL-37 requires the receptors IL-18Rα and IL-1R8 (SIGIRR) to achieve its extracellular functions [41]. Jiang et al. reported that serum IL-37 abundance is decreased in Rcc patients and negatively correlates with the disease progression [21]. Although our study only describes the intracellular activity of IL-37b on the Smad3 signaling in HCC, it is conceivable that IL-37b secretion by liver nonparenchymal cells may also play an anticancer effect via extracellular signal transduction. Examination of this hypothesis and confirmation of the specific molecular mechanism will be the focus of our future studies. Deeper knowledge of the risk factors associated with the hepatocarcinogenesis can improve the effectiveness of HCC surveillance programs. It has been shown that Smad3 phosphorylation may be a prognostic risk factor indicator in the progression of human chronic liver diseases [26]. In this study, we found a negative correlation of IL-37b with pSmad3L in HCC tissue samples. These data suggest that evaluation of the IL-37b-pSmad3L axis as a new prognostic marker in patients with HCC may be crucial because not only do they provide a new criterion for prognosis, but may also be a potential therapeutic target.

In summary, we have determined that IL-37 is down-regulated in HCC. IL-37b potentially possesses the efficiency to suppress HCC growth, at least in part, through the conversion of Smad3 phospho-isoform signaling from JNK/pSmad3L/c-Myc oncogenic signaling to pSmad3C/p21 tumor-suppressive signaling. Therefore, IL-37 could function not only as a fundamental inhibitor of innate immunity, but also as a novel tumor suppressor in HCC. The identification of IL-37 and its crosstalk within different oncogenic pathways in HCC progression may help in a better understanding of therapeutic strategy for HCC or other cancers.

## MATERIALS AND METHODS

### Cell lines and culture conditions

Normal liver cell lines LO2 and QSG-7701; and liver cancer cell lines SMMC-7721, HepG2, Huh7, and MHCC-97H were obtained from Cell Bank of Type Culture Collection of Chinese Academy of Sciences, Shanghai Institute of Cell Biology. Cell lines were maintained in RPMI 1640 medium supplemented with 10% FBS and 1% penicillin/streptomycin (Gibco, Life Technologies, USA) in a humidified 37 °C incubator with an atmosphere of 5% CO_2_.

### Patients and tissue specimens

A total of 101 paired HCC and adjacent non-cancerous hepatic tissues were obtained from patients during surgical HCC resection at the First Affiliated Hospital Hepatobiliary Surgery Department of Chongqing Medical University, P.R. China from January 2009 to January 2010. All HCC specimens were confirmed by pathological examination. None of these patients had received pre-operative chemotherapy or radiotherapy. The post-operative follow-up occurred at the outpatient department within the hospital. Overall survival (OS) was defined as the period from the surgery to death or the last known follow-up. Disease-free survival (DFS) was defined as the period from the surgery to recurrence or the last follow-up if no recurrence was observed. This study protocol was approved by the Institute Research Ethics Committee of the First Affiliated and all patients provided informed consent for the study to retain and analyze their tissues for research purposes.

### Immunohistochemistry (IHC) analysis

IHC for protein expression in HCC and adjacent non-cancerous hepatic tissues were performed using specific antibodies. Briefly, sections were de-paraffinized, subjected to microwave antigen retrieval for 15 min in sodium citrate solution (pH 6.0) and then incubated with 3% hydrogen peroxide to block endogenous peroxidase activity. The sections were incubated overnight at 4°C with primary monoclonal antibodies, including mouse anti-human IL-37 (Abcam:ab57187, USA; dilution 1/250), rabbit anti-human phospho-Smad3 (S204) (ImmunoWay:YP0360, USA; dilution 1/250). After incubation with the primary antibody, the sections were incubated with appropriate secondary antibodies(Santa Cruz Biotechnology, USA;dilution 1/1000,) at 37°C for one hour. Finally, DAB(beyotime, beijing, China) was used for signal development, counterstained with hematoxylin. Sections stained with PBS only were used as the negative staining control. Under high-power magnification (×200, ×400), images of three representative fields were captured by the Leica QWin Plus v3 software; identical settings were used for each photograph. The density was counted by Image-Pro Plus v6.2 software (Media Cybernetics Inc, Bethesda, MD). For the reading of each antibody staining, a uniform setting for all the slides was applied. Integrated optical density(IOD) of all the positive staining in each photograph was measured, and its ratio to total area of each photograph was calculated as density, The detail was performed as previously described [23]. Based on the Integrated optical density(IOD) of all 101 patients. IOD values are arranged from low to high.No.1-No.51 were defined as low expression group.No.52-No.101 were defined as low expression group.

### Vector construction and transfection

Human *IL1F7b*-recombined lentiviral expression vector and the control vector were purchased from GeneChem (Shanghai, China). The *IL1F7b* vectors were constructed by inserting their ORF sequence into the GV358 (GeneChem, Shanghai, China) vector with expression sequence encoding green fluorescent protein(GFP). Transfection was performed according to the manufacturer's protocol. We designated lentiviral vectors encoding the human *IL1F7b* gene or GFP as LV_*IL1F7b* or LV_Negative control(LV_NC). Viruses were harvested 72 h after transfection and viral titers were 1×109 TU/mL;Human HCC cell line SMMC-7721 and HepG2 cells were infected with lentivirus (LV_*IL1F7b* or LV_NC) at a multiplicity of infection (MOI) of 10 in the presence of 5 mg/ml polybrene (GeneChem, Shanghai) and cultured in Enhanced Infection Solution (ENi.S.)(GeneChem, Shanghai). After infection for 12 h, cells were maintained in RPMI 1640 supplemented with 10% FBS. The cells were selected using 5 mg/ml puromycin and screened for stable cell lines. IL1F7-specific siRNA were purchased from Genepharma (Shanghai, China), and 100 nM comprised 25 nM of the four following antisense sequences: (I), ucaaggaugaggcuaaugcuu; (II), caauguguuuccuguucucuu; (III), uuacaauugcaggaggugcuu; (IV), uuauccuugucacaguagauu (all 5' to 3'). Transfections were performed with a Lipofectamine 2000 kit (Invitrogen, Carlsbad, CA) according to the manufacturer's instructions. Transfected cells were harvested at 48 h.

### Reverse transcription and qRT-PCR

RNA was extracted using TRIzol reagent (TaKaRa, Dalian, China), and reverse transcription was performed using the RevertAid First Strand cDNA Synthesis kit (Thermo Scientific, USA). Real-time PCR was performed with SYBP Premix Ex Taq (TaKaRa, Dalian, China) according to the manufacturer's protocol. The primers for IL-37b and GAPDH were as follows:IL-37b:forward:5’-GATCACAAAGTACTGGTCCTGG-3’, reverse: 5’-TCCTTTATCCTTGTCACAGTAG-3’;GAP DH:forward5’-GAAGGTCGGAGTCAACGGATTT-3’, reverse:5’-CCTGGAAGATGGTGATGGGATT-3’. The expression level of housekeeping gene GAPDH using the comparative threshold cycle (2^-ΔΔCt^) method.IL-37 was normalized to the expression of the qRT-PCR reactions were performed in triplicate and included no-template controls. Relative expression was calculated with the 2^-ΔΔCt^ method.

### ELISA, and western blot analysis

LV_*IL1F7b* or LV_NC-infected SMMC-7721 cells were starved for 15 h in serum-free medium and were incubated for 8 h in the absence or presence of 10 μM JNK inhibitor SP600125 (MedChem Express, USA). The cells were then treated with 400 pM tumor necrosis factor (TNF)-α (R&D Systems) or isodose Phosphate Buffered Saline (PBS) for 30 mins. As previously described, cells were incubated for 8 h in the absence or presence of 2 μM Smad3 activation inhibitor SIS3 [24], then treated with 20 pM TGF-β1 (R&D Systems) or isodose PBS. The levels of TGF-β, IL-6, IL-8, IL-1β, TNF-α, and MMP2 in culture supernatants were measured by ELISA, following the manufacturer's instructions (R&D Systems). After stimulation at indicated time-points, cell supernatants were collected and analyzed using a Quantikine ELISA Kit (R&D Systems) according to the manufacturer's Instructions. Western blotting was performed as previously described [25]. The specific primary antibodies human IL-37, JNK1+JNK2+JNK3, c-myc, p21, cdc2, cyclinB1, cyclinA2, cyclinD1 (Abcam, Cambridge, MA) and Smad3, p-smad3(s204), p-smad3(s425) (Immunoway, Newark, USA), p-JNK(Thr183/Tyr185), and β-actin(Cell Signaling Technology) were used.

### Cell proliferation, cell cycle analysis, cell apoptosis, and colony formation assays

The effect of IL-37 on HCC cell growth was determined with CCK-8 assays. After 48 hour transfection, cells were seeded into 96-well flat-bottom plates (1×10^3^/well) then cultured for 1 to 4 days in medium using a CCK-8 Kit (Dojindo, Kumamoto, Japan) according to the manufacturer's protocol. For cell cycle analysis, after 48 hour transfection, a total number of 5×10^5^ cells were washed with PBS and fixed with ice cold 70% ethanol in PBS at 4°C overnight. Then, fixed cells were treated with DNA-staining solution (3.4 mmol/L Tris-Cl (pH 7.4), propidium iodide (PI), 0.1% Triton X-100 buffer, and 100 mg/mL RNase A). Stained cells were subjected to fluorescence-activated cell sorting (FACS) flow-cytometry analysis of cells percentage in each phase of the cell cycle. For cell apoptosis assays, The flow cytometry assay was performed to determine the levels of early-stage apoptosis, using ApoScreen Annexin V kit (BD Biosciences, USA) according to the manufacturer's protocol. Briefly, HCC cells were digested by 0.25% trypsin-EDTA, washed by cold PBS one time and resuspended in cold binding buffer at concentrations between 1×10^5^ and 1×10^6^ cells/ml. 5μL of labeled Annexin-V was added into 100 μL of the cell suspension. After incubation on ice for 15 min, 200 μL binding buffer and 5 μL 7-AAD solution were added into the cell suspensions. The number of stained cells was assessed immediately by FACS Calibur (BD). For colony formation assays, transfected cells were seeded in 6-well plates at 1,000 cells per well. Two weeks later, the colonies were fixed with 4% paraformaldehyde and stained with a crystal violet solution for 10 min. Cell colonies were counted using Image-Pro Plus 6.0 software. All studies were conducted in triplicates.

### Tumorigenicity in BALB/c nude mice

All the animal studies were approved by the Animal Ethics Committee of Chongqing Medical University. Female 6-week-old BALB/c nude mice were purchased from Institute of Laboratory Animal Science (Chinese Academy of Medical Science, Beijing, China). For the tumor growth model, 1×10^6^ LV_*IL1F7b* or LV_NC infected SMMC-7721 cells were injected subcutaneously into nude mice and tumor growth was monitored each week (n=5/group). The mice were sacrificed on day 35 after tumor implantation. Tumor tissues were prepared for Western blot analysis.

### Statistical analysis

All *in vitro* experiments were performed in triplicate and independently repeated three times. The differences in IL-37 or pSmad3L expression between HCC and adjacent non-cancerous tissues were assessed by the Mann-Whitney U-test. The relationships between IL-37 and clinicopathological features were evaluated by the χ2 test. The survival rates in relation to IL-37 expression were estimated by the Kaplan-Meier method, and the difference in survival curves was tested by the log-rank test. Statistical differences in cell proliferation and invasion assays were determined by the Student's t test (two-tailed). The relationship between IL-37 and pSmad3L expression was explored by the Spearman rank correlation. The statistical differences between three groups or more groups were assessed by the one-way ANOVA followed by LSD and S–N–K Test. The SPSS 19.0 statistical software was used to perform all statistical analyses. Data are presented as the means±standard deviation (SD). P < 0.05 was considered statistically significant.

## SUPPLEMENTARY MATERIALS FIGURE



## Abbreviations

IL-37: interleukin 37
HCC: hepatocellular carcinoma
JNK: c-Jun N-terminal kinase
pSmad3C: C-terminally phosphorylated Smad3
pSmad3L: linkerphosphorylated Smad3
OS: overall-survial
DFS: disease-free survival
TGF-β: transforming growth factor
TβRI, TGF-β: type I receptor
