# Supplementary material for: IL-37 suppresses hepatocellular carcinoma growth by converting pSmad3 signaling from JNK/pSmad3L/c-Myc oncogenic signaling to pSmad3C/P21 tumor-suppressive signaling
Source: Oncotarget. 2016 Nov 8;7(51):85079–96. doi: 10.18632/oncotarget.13196 (PMC5356721; doi:10.18632/oncotarget.13196)
Supplement: Supplementary file 1 [file oncotarget-07-85079-s001.pdf]

IL-37 suppresses hepatocellular carcinoma growth by converting pSmad3 signaling from JNK/pSmad3L/c-Myc oncogenic signaling to pSmad3C/P21 tumor-suppressive signaling

SUPPLEMENTARY FIGURE

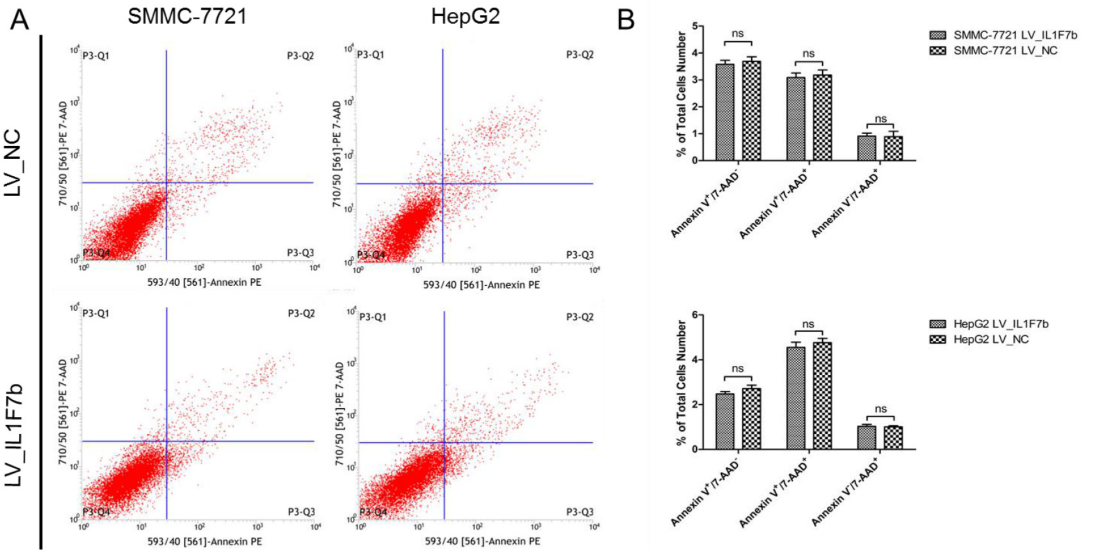

Supplementary Figure S1: IL-37b has no effects on HCC cells apoptosis *in vitro*. A. FACS-based Annexin V/7-AAD assay showed that overexpression LV_*IL1F7b* has no effects on the number of both Annexin V^+/7-AAD^-(early-stage apoptosis) and Annexin V^+/7-AAD^+ (late-stage apoptosis) cells; In the four felds of the original images from the FACS-based study, the number of the dots in the bottom-left feld, the bottom-right feld, the top-left feld and the top-right feld indicates the number of Annexin V^-/7-AAD^-, Annexin V^+/7-AAD^-, Annexin V^-/7-AAD^+, and Annexin V^+/7-AAD^+ cells, respectively. **B.** Quantifcations of the results from the FACS-based Annexin V/7-AAD assay showed that overexpression LV_*IL1F7b* has no effects on the number of both Annexin V^+/7-AAD^-(early-stage apoptosis) and Annexin V^+/7-AAD^+ (late-stage apoptosis) cells. Data were collected from three independent experiments. $^{ns}P>0.05$.
